# Self-Learning Multimodal Emotion Recognition Based on Multi-Scale Dilated Attention

**DOI:** 10.3390/brainsci16040350

**Published:** 2026-03-25

**Authors:** Xiuli Du, Luyao Zhu

**Affiliations:** 1 School of Information Engineering, Dalian University, Dalian 116622, China; zhuluyao@s.dlu.edu.cn; 2 Communication and Network Laboratory, Dalian University, Dalian 116622, China

**Keywords:** EEG, facial expressions, multi-scale dilated convolution, multimodal, emotion recognition, action units, differential entropy, decision-level fusion

## Abstract

**Highlights:**

**What are the main findings?**

- We propose a Multi-Scale Dilated Attention Convolution network specifically designed for facial expression; it efficiently captures both local micro-expression details and global semantic features, leading to substantially improved facial emotion recognition performance.
- We introduce a self-learning weight fusion mechanism for decision-level fusion of EEG and facial modalities; compared with fixed or simple weighted fusion, this method adaptively selects the optimal fusion weighting and yields superior multimodal classification results.

**What is the implication of the main finding?**

- The proposed method was separately evaluated on the FER2013, CK+, and DEAP datasets, where it demonstrated superior performance over existing approaches in facial single modal, EEG single modal, and multimodal fusion settings.

**Abstract:**

Background/Objectives: Emotions can be recognized through external behavioral cues and internal physiological signals. Owing to the inherently complex psychological and physiological nature of emotions, models relying on a single modality often suffer from limited robustness. This study aims to improve emotion recognition performance by effectively integrating electroencephalogram (EEG) signals and facial expressions through a multimodal framework. Methods: We propose a multimodal emotion recognition model that employs a Multi-Scale Dilated Attention Convolution (MSDAC) network tailored for facial expression recognition, integrates an EEG emotion recognition method based on three-dimensional features, and adopts a self-learning decision-level fusion strategy. MSDAC incorporates Multi-Scale Dilated Convolutions and a Dual-Branch Attention (D-BA) module to capture discontinuous facial action units. For EEG processing, raw signals are converted into a multidimensional time–frequency–spatial representation to preserve temporal, spectral, and spatial information. To overcome the limitations of traditional stitching or fixed-weight fusion approaches, a self-learning weight fusion mechanism is introduced at the decision level to adaptively adjust modality contributions. Results: The facial analysis branch achieved average accuracies of 74.1% on FER2013, 99.69% on CK+, and 98.05% (valence)/96.15% (arousal) on DEAP. On the DEAP dataset, the complete multimodal model reached 98.66% accuracy for valence and 97.49% for arousal classification. Conclusions: The proposed framework enhances emotion recognition by improving facial feature extraction and enabling adaptive multimodal fusion, demonstrating the effectiveness of combining EEG and facial information for robust emotion analysis.

## 1. Introduction

Emotion, as a comprehensive manifestation of human psychological and physiological states, not only integrates perception, cognition, and behavioral responses but also serves as a core nexus for the interaction between individuals and their environment. Accurate emotion recognition holds significant importance across various domains, including healthcare, smart homes, autonomous driving, commerce, and nursing companionship [1]. Existing emotion recognition research can be divided into two categories according to the type of signals collected: emotion recognition based on behavioral manifestations and emotion recognition based on neurophysiological signals. Emotion recognition based on behavioral manifestations includes the recognition of external behaviors such as posture and gestures, speech and intonation, and facial expressions. Emotion recognition based on neurophysiological signals refers to the recognition of internal neurophysiological signal changes, including Electroencephalogram (EEG) [2], Electrocardiogram (ECG) [3], and Galvanic Skin Response (GSR) [4].

While behavioral signals are easily acquired and intuitively interpretable, they possess an inherent limitation of highly susceptible to subjective manipulation [5]. Individuals may mask their true emotional states due to social norms or emotion regulation strategies, leading to a significant decline in recognition accuracy in non-cooperative scenarios. In contrast, neurophysiological signals are directly regulated by the autonomic nervous system and possess the advantages of anti-disguise capabilities and objective authenticity [6]. However, they face technical bottlenecks such as stringent acquisition conditions, low signal-to-noise ratios, and substantial individual variability.

Compared with single-modal emotion recognition, multimodal emotion recognition can explore the consistency and complementary features of emotional data from different modalities. Fully fusing this information enables machines to better comprehend human emotions [7,8,9]. Among these, the multimodal fusion of EEG signals and facial expressions is particularly critical. Compared with behavioral modality signals, EEG signals demonstrate notable advantages in terms of resistance to deliberate falsification, high temporal resolution, and rich informational content, thereby furnishing a more objective and reliable physiological basis for emotion recognition [2]. Facial expressions, on the other hand, constitute one of the most natural and intuitive channels through which humans convey emotional states and intentions, and a considerable body of research methodologies dedicated to facial expression-based emotion recognition has been established [10]. Based on the advantages of these two modalities, combining EEG signals with facial expressions can, to a certain extent, improve the recognition rate of emotions [11,12].

However, existing research in multimodal emotion recognition still faces two fundamental challenges. On the one hand, facial Action Units (AUs) are not consistently continuous; furthermore, the distances between different AUs vary, and they contribute differently to emotional expression. Traditional convolutional neural networks struggle to simultaneously capture fine-grained local micro-expression details and global semantic information in facial emotion recognition, as their inherent fixed receptive fields limit representational flexibility. On the other hand, the modeling of cross-modal decision interaction is inadequate, since most existing approaches rely on simple concatenation or fixed-weight fusion without explicitly characterizing the interaction between EEG and facial expression classification decisions. As a result, the contribution of each modality cannot be adaptively adjusted under different emotional states, thereby limiting the effectiveness of multimodal fusion.

To address these issues, this study proposes a self-learning multimodal emotion recognition method based on multi-scale dilated attention. This paper proposes two solutions to address the inherent limitations of current EEG and facial expression-based multimodal emotion recognition methods in capturing spatially discontinuous facial cues and modeling modality-specific decision confidence. Specifically:

1. A Multi-Scale Dilated Attention Convolutional Network (MSDAC) is designed for facial emotion recognition. This network innovatively integrates a Multi-Scale Dilated Convolution module (MSDC) with a Dual-Branch Attention module (D-BA) to simultaneously capture local facial texture and global structural information. The Dual-Branch Attention module enhances feature responses in emotionally significant regions (such as the eyes and mouth), significantly improving micro-expression discrimination capability while maintaining a lightweight design.
2. A self-learning multimodal fusion strategy is proposed, beyond static weighting by explicitly characterizing the dynamic reliability of heterogeneous signals. Taking single-modal classification probabilities as input, this strategy utilizes a multi-head self-attention mechanism to explicitly model the cross-modal dependencies between EEG and facial decisions. By adaptively learning the collaborative decision patterns of the two modalities, it more fully mines modal synergistic information compared to simple concatenation or fixed-weight fusion, thereby achieving complementary advantages at the decision level.

## 2. Related Work

Facial Emotion Recognition (FER) has currently proposed many research methods. Mu et al. [13] proposed a deep learning method based on texture features, which simplifies the learning process of CNN by leveraging the low-level information of texture images, significantly reducing the model training time. Yang et al. [14] proposed LightExNet, which solves the problems of blind feature extraction and insufficient mining of spatial dimension information in expression recognition by the MobileNet V2 method through the fusion of deep and shallow feature channels, the improved channel-spatial attention mechanism and the optimized center loss function. Facial expressions are composed of multiple distinct facial AUs, each contributing differently to emotional expression. The relative importance of these AUs varies, and how to enable the network to focus more on these key features is a crucial research direction for improving emotion recognition accuracy. Mi et al. [15] proposed a deep attention convolutional network, DeepEmotion, which consists of fewer than 10 layers. They introduced a spatial transformation network to direct attention to the key regions of the human face. Fan et al. [16] presented the AttenVGG model, which integrates the attention mechanism with the VGG19 architecture, enhancing the performance of expression recognition. Che et al. [17] proposed a spatial and channel attention CNN (SCA-CNN) that combines spatial and channel attention for image captioning. Woo et al. [18] proposed the Convolutional Block Attention Module (CBAM), a sequential module that uses a spatial attention submodule and a channel attention submodule. Ordinary neural networks are often unable to capture the correlation of Action Units (AUs) at varying distances, and the dynamic changes in facial expressions frequently depend on the synergy of AUs across different regions. Some studies have addressed this issue by employing multi-scale convolutional networks to extract information from regions of various sizes. Zhao et al. [19] proposed a global multi-scale local attention network for facial emotion recognition in uncontrolled environments, which integrates global multi-scale features with local attention features, effectively addressing challenges such as occlusion and non-frontal poses. Li et al. [20] introduced a lightweight facial expression recognition method that combines the Swin Transformer with a multi-scale feature fusion module. Ni et al. [21] leveraged feature maps at different levels within a pyramid convolutional structure to capture multi-scale information for facial expression recognition. Luo et al. [22] proposed a Multi-scale Integrated Attention Network, which incorporates the Inception structure, the ECA attention mechanism, and depth wise separable convolutions, achieving both effective feature extraction and a lightweight network design. While the aforementioned methods effectively extract multi-scale features, they often rely on increasing network depth or employing complex feature pyramid structures. This reliance results in a significant increase in computational cost and limits the processing to features from continuous facial regions. To address the insufficiency of existing methods in capturing non-continuous, cross-region AU associations, as well as the tendency of standard convolutions to introduce redundant information, this paper proposes the MSDAC. Unlike approaches that stack convolutional layers, MSDAC utilizes multi-scale dilated convolutions to expand the receptive field without increasing the number of parameters, thereby precisely capturing long-range AU associations. Furthermore, by integrating an improved Dual-Branch Attention mechanism, the network filters out background redundancy and achieves precise focus on key micro-expression regions.

Currently, the primary research focus of EEG-based emotion recognition has shifted to how to better leverage multi-dimensional features. Ozdemir et al. [23] first converted raw EEG signals into topological structures corresponding to the positions of the acquisition electrodes. They then employed convolutional neural networks (CNN) to extract spatial information and long short-term memory networks (LSTM) to capture hidden temporal information embedded in the features, achieving high recognition accuracy on the arousal and valence dimensions of the DEAP dataset. Hu et al. [24] proposed a spatio-temporal adaptive fusion network, which captures brain connectivity patterns through spatial dynamic evolution and incorporates a multi-structure Transformer-based fusion module to effectively integrate the temporal and spatial characteristics of EEG signals. An et al. [25] fused the differential entropy (DE) features of different EEG frequency bands while preserving the spatial relationships among channels, and subsequently employed a convolutional autoencoder for the emotion recognition classification task, further improving the overall classification performance. The aforementioned studies indicate that combining any two of the three feature dimensions—time, spatial, and frequency domains—in EEG signals can effectively enhance the accuracy of emotion recognition and classification. Recently, several studies have proposed network models capable of simultaneously extracting information from the time, spatial, and frequency domains [26,27]. These studies indicate that combining time, spatial, and frequency dimensions enhances accuracy. However, efficient integration of these features within a multimodal framework remains a challenge, as complex single-modal models may lead to computational bottlenecks. In this paper, we employ a streamlined feature extraction strategy in the EEG branch to ensure a robust and efficient decision basis for subsequent multimodal fusion.

The information contained within a single modality is inherently limited, and incorporating data from multiple modalities can yield more comprehensive emotional information. According to existing studies, modal fusion methods can be broadly categorized into four types: data-level fusion, feature-level fusion, decision-level fusion, and model-level fusion [28]. Wang et al. [29] integrated facial features with the spatial and channel features of EEG signals for multimodal emotion recognition. Salama et al. [30] proposed a three-dimensional convolutional neural network architecture to extract spatio-temporal features from EEG and facial video data, and generated the final fused prediction by combining data augmentation with ensemble learning techniques. However, given the significant heterogeneity in data across different modalities, it is essential to select models tailored to each specific modality. Decision-level fusion enables each modality to employ optimal preprocessing, feature extraction, and classification methods independent of other modalities, thereby ensuring strong model independence. Furthermore, due to this independent processing, the recognition results from other modalities remain utilizable even if the data quality of a specific modality is compromised or missing. Zhang et al. [31] proposed the PMD algorithm, which achieves emotion recognition via decision-level weighted fusion of EEG and peripheral physiological signals, with fusion weights determined through an offline grid search. Existing decision-level fusion methods predominantly employ fixed-weight addition or voting mechanisms, often overlooking the variations in confidence levels across modalities under different emotional states. Such rigid fusion strategies limit the utilization of inter-modal complementarity. To address this, this paper proposes a self-learning decision-level fusion method based on multi-head attention mechanisms, which explicitly models deep dependencies between EEG and facial decisions to adaptively learn optimal fusion weights.

This paper proposes a multimodal emotion recognition model based on EEG signals and facial expressions. The model performs emotion recognition on facial expressions and EEG signals through two parallel network branches, subsequently fusing the outputs of the two modalities at the decision level to obtain the final classification result. To address the heterogeneity and discontinuity in the distances between individual AUs in facial expressions, as well as the inability of conventional neural networks to accurately capture key facial action units related to emotional expression, the facial recognition branch employs an MSDAC for emotion recognition. This network comprises two components. The MSDC module adjusts the receptive field size through different dilation rates, thereby extracting AU information at multiple scales and distances. The D-BA integrates channel and spatial attention mechanisms to further enhance the representational capacity of key features, enabling the model to focus on local facial regions that are more critical for emotion recognition. Emotion recognition based on EEG signals employs a time–frequency–spatial feature extraction approach and incorporates a global attention mechanism to effectively capture the multidimensional information contained in EEG signals. To address the issue that the confidence levels of each modality differ during decision-level fusion and that conventional fusion methods may result in rigid allocation, a self-learning weight module is designed to dynamically fuse the outputs of the two modalities. This module automatically learns the optimal fusion strategy for different emotion categories, thereby improving the final recognition performance.

## 3. Proposed Method

**Figure 1:**
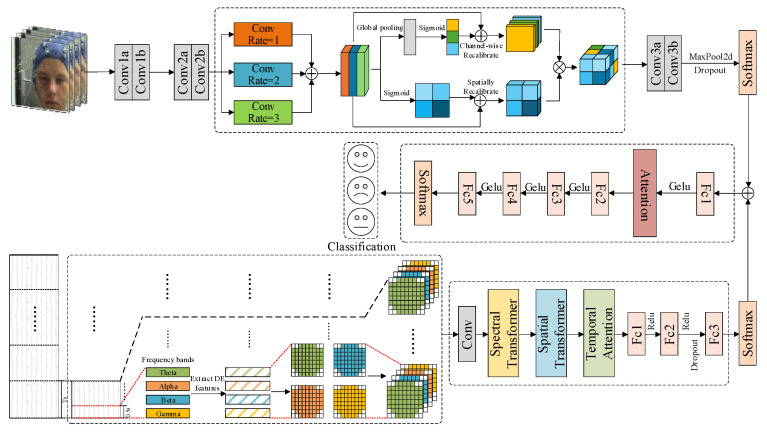
Diagram of the multimodal emotion recognition model. The proposed model takes EEG signals and facial expression images as inputs, and generates binary classification outputs across two affective dimensions: the valence dimension (high valence/low valence) and the arousal dimension (high arousal/low arousal). All emotional states can be conceptualized as combinations of varying degrees of arousal and valence.

The overall structure of the multimodal emotion recognition model proposed in this paper is illustrated in Figure 1. The model consists of three components: the facial emotion recognition branch, the EEG emotion recognition branch, and the self-learning weight-based decision fusion module.

### 3.1. Facial Expression Emotion Recognition Based on Multi-Scale Dilated Attention Convolution

**Figure 2:**
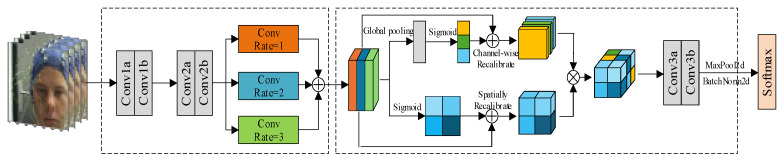
Module Structure Diagram of MSDAC.

The design of MSDAC is motivated by the fact that emotional facial expressions are not localized to a single region but involve the synergy of distant AUs. The AU combinations corresponding to different emotions exhibit significant differences in spatial scale. For example, happiness is primarily composed of the contraction of the zygomaticus major (AU6) and the upward pull of the lip corners (AU12), resulting in a relatively compact spatial distribution. Fear, by contrast, simultaneously involves the inner brow raise (AU1), brow lowerer (AU4), and lateral lip stretcher (AU20), among other AUs distributed across multiple facial regions, resulting in a spatial span far greater than the former. This physiological fact suggests that convolutional kernels with a single fixed receptive field have a structural limitation in extracting features across different emotion categories. Unlike traditional multi-scale approaches that rely on depth to expand receptive fields, our use of dilated convolutions allows the network to bridge these non-continuous spatial gaps with minimal computational cost. The multi-scale dilated attention network is designed to enhance feature representation by employing multiple dilation rates and integrating both channel and spatial attention mechanisms, as depicted in Figure 2. The facial images are first preprocessed to localize the facial region and remove irrelevant background information. The preprocessed images are then passed through two convolutional blocks to extract global low-level features. Subsequently, a parallel three-branch convolutional module is constructed to facilitate multi-scale feature extraction. The three parallel branches in this paper are designed with clear physiological correspondence. The first branch has the smallest receptive field with a dilation rate of 1, focusing on capturing local fine-grained features such as micro-expression cues including eye corner wrinkles and lip corner curvature. The second branch has a moderate receptive field with a dilation rate of 2, suited for encoding mid-range AU synergies such as the coordinated movement between the eyebrows and eyelids. The third branch has the largest receptive field with a dilation rate of 3, capable of perceiving large-scale muscle group structures across facial regions, such as the coordinated activation of the zygomaticus major and the orbicularis oris. The complementary parallelism of the three scales enables the model to simultaneously respond to the complete spatial hierarchy of emotional expression, from local to global.

All three branches employ 3 × 3 convolutional kernels, which enlarge the receptive field without increasing the number of parameters, thereby enabling the extraction of spatial emotional information across different ranges and accommodating the multi-scale variation and distribution of facial expression features. Traditional convolutional networks typically rely on stacking multiple convolutional layers to expand the receptive field, which not only increases computational cost but may also dilute informative features. In contrast, dilated convolutions enlarge the receptive field by adopting different dilation rates while preserving the resolution of the feature maps. Since facial action units exhibit variations in size and shape, utilizing multiple parallel dilation rates allows the network to capture features at diverse spatial scales simultaneously. This design enhances the model’s adaptability and discriminative capability for multi-scale facial expression characteristics.

Traditional convolutional operations fail to dynamically adjust the importance of channels and treat all spatial positions equally, thereby being unable to highlight key regions. For example, the emotional information conveyed by the eyes is typically more informative than that conveyed by the cheek muscles. Therefore, after extracting multi-scale features from the three branches, these features are concatenated along the channel dimension to form an aggregated feature map, which is subsequently refined by the dual-branch attention module designed in this study. Channel attention first applies global average pooling to the aggregated feature map to obtain a global feature of each channel. It then learns the importance weight of each channel through two fully connected (FC) layers and performs channel-wise multiplication with the original feature map to achieve adaptive channel reweighting. The spatial attention mechanism performs global pooling on the aggregated feature map along the channel dimension to obtain the spatial feature maps. Through a 1 × 1 convolution followed by a sigmoid activation function, the importance of each spatial position is learned, thereby enabling spatial-wise reweighting. Finally, the outputs of the dual-branch attention module are fused to produce the final enhanced feature maps. The enhanced facial features are passed through the final convolutional block, a max-pooling layer, and a batch normalization layer before being fed into the FC layer for emotion recognition. To mitigate overfitting, a dropout layer with a dropout rate of 0.2 is applied subsequent to the first fully connected layer within the classifier.

### 3.2. Emotion Recognition for EEG Based on Three-Dimensional Features

To fully utilize the temporal, frequency, and spatial dimensional features in EEG signals, this paper constructs a three-dimensional feature structure for EEG. First, the original EEG signal is divided into N segments of equal length with each segment lasting T seconds. Each segment is then decomposed into four frequency bands—θ [4–7 Hz], α [8–13 Hz], β [14–30 Hz], and γ [31+ Hz]—using band-pass filtering. DE, which can reflect the rate and trend of changes in EEG signals, is the most representative feature in the frequency domain. Therefore, for each signal segment in each frequency band, we calculate the DE feature with a 0.5-s time window. The mathematical expression of differential entropy is as follows:

(1) $$h(x)=-\int_{-\infty }^{\infty }f(x)\mathrm{lg}[f(x)]dx$$

where x is formally a random variable and in this context, the signal acquired from a certain frequency band on a certain EEG channel, f(x) is the probability density function of x. If x obeys the Gaussian distribution $N(\mu ,\sigma_{i}^{2})$, where $\sigma_{i}^{2}$ is the variance and μ is the signal mean, DE feature can simply be calculated by the following formulation:

(2) $$h(x)=\int_{-\infty }^{\infty }\frac{1}{\sqrt{2\pi \sigma_{i}^{2}}}e^{\frac{{\left(x-\mu \right)}^{2}}{2\sigma_{i}^{2}}}\mathrm{lg}[\frac{1}{\sqrt{2\pi \sigma_{i}^{2}}}e^{\frac{{\left(x-\mu \right)}^{2}}{2\sigma_{i}^{2}}}]dx$$

**Figure 3:**
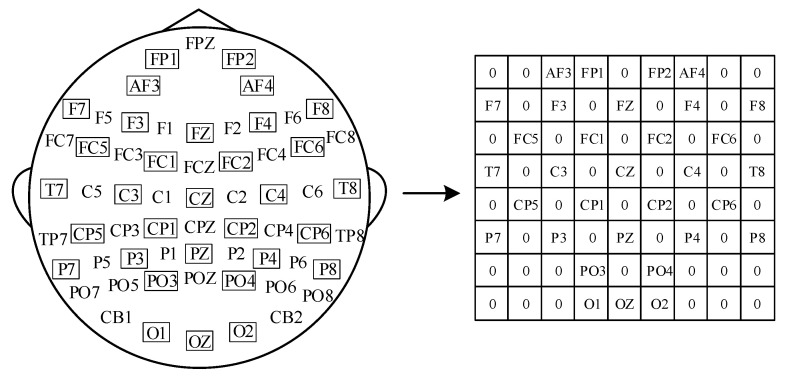
EEG Electrode Position Map.

First, based on the relative positions of the 32-channel electrodes, the DE feature vector is transformed into a compact 2D map by constructing an 8 × 9 two-dimensional matrix, where positions without electrodes are filled with zeros, as shown in Figure 3. Then, the 2D DE feature maps of different frequency bands are stacked together to obtain a 4 × 8 × 9 three-dimensional feature matrix.

The input data has a shape of (batch size, 6, 4, 8, 9), which includes 6 times steps, 4 channels, and an 8 × 9 spatial dimension. To adapt to subsequent processing, the data is reshaped and fed into a 2D convolutional layer. Spectral attention and spatial attention modules are used to process spectral features and spatial features, respectively. Both modules adopt a single-layer multi-head self-attention Transformer encoder (L = 1) equipped with 4 attention heads and an embedding dimensionality of 72, thereby better accommodating the 4-band, 32-channel input dimensionality of the DEAP dataset. The multi-head attention mechanism can simultaneously extract information from multiple subspaces and dynamically assign weights through the self-attention mechanism to more accurately capture the key features in the input data. Subsequently, the temporal attention mechanism generates a scalar weight for each time step through a linear projection followed by a ReLU activation function, after which a weighted summation is computed along the temporal axis to produce a fixed-length representation. The EEG signals output multidimensional temporal-frequency-spatial feature representations through global attention modules for spectral, spatial, and temporal dimensions, and obtain final emotional classification results via a fully connected layer (input dimension 1728 = 6 × 4 × 8 × 9, output dimension 2) with a dropout rate of 0.5 applied after the first layer.

### 3.3. Self-Learning Weight Module

To effectively fuse modalities with varying importance and complementary characteristics, and to prevent low-confidence modalities from negatively affecting the final accuracy, this paper proposes a self-learning weight module for dynamic decision-level fusion of EEG and facial expression data. The core advantage of this module lies in its ability to automatically learn and adjust the weight of each modality based on the input data during the training process. This allows it to adaptively optimize the decision fusion strategy in different emotion classification scenarios, overcoming the limitations of traditional fixed-weight fusion methods, which lack flexibility.

The probability distribution outputs of facial expressions and EEG signals serve as the input for decision-level fusion, which are weighted and fused through the designed Self-Learning Weight Module. During this process, the probability distributions of each modality are concatenated into a large feature vector, whose dimensions are gradually expanded through an FC layer while introducing nonlinear activation functions GELU. Assume the probability distribution output of EEG signals is $X_{E}$ and that of facial expressions is $X_{F}$, both with dimensions [N,D], where N is the number of samples and D is the feature dimension. The features of the two modalities are concatenated along the feature dimension to obtain the fused feature representation $X_{fused}$:

(3) $$X_{fused}=(X_{E},X_{F})\in {\mathbb{R}}^{N\times 2D}$$

The model applies non-linear transformations to the fused features through a multi-layer FC:

(4) $$H_{i}=GELU(H_{i-1}W_{i}+b_{i})$$

where $W_{i}\in {\mathbb{R}}^{2D\times N}$ and $b_{i}\in {\mathbb{R}}^{N}$ are the weight and bias of the i-th layer (i = 1,2,3,4), $H_{i}$ is the output of the i-th layer (i = 1, 2, 3, 4) and $H_{0}=X_{fused}$. Reshape the features into a sequence form suitable for the attention mechanism:

(5) $$X_{seq}=reshape(H_{i},[N,L,E])$$

where L represents the sequence length, E denotes the embedding dimension, and $X_{seq}$ is the reshaped output. Subsequently, the processed data is passed through a multi-head self-attention layer, where multiple attention heads simultaneously perform adaptive weighting on the feature sequence. Each attention head independently computes the linear transformations of the Queries, Keys, and Values, thereby dynamically learning the relative contribution weights to emotion classification. For each attention head h∈{1,…,n}, the Query $Q_{h}$, Key $K_{h}$, and Value $V_{h}$ matrices are computed as:

(6) $$Q_{h}=X_{seq}S_{h}^{Q}$$

(7) $$K_{h}=X_{seq}S_{h}^{K}$$

(8) $$V_{h}=X_{seq}S_{h}^{V}$$

where $S_{h}^{Q}$, $S_{h}^{K}$ and $S_{h}^{V}$ are the learnable parameter matrices for the h-th head. By using the attention score matrix to perform weighted summation on the value matrix, the output of a single head $head_{h}$ is:

(9) $$head_{h}=soft\;\max(\frac{Q_{h}K_{h}^{T}}{\sqrt{d_{k}}})\cdot V$$

where $d_{k}$ The outputs of all attention heads are concatenated and linearly transformed to obtain the final multi-head self-attention output $X_{attn}$:

(10) $$X_{attn}=(head_{1},\ldots ,head_{n})W^{O}$$

where $W^{O}$ is the linear transformation matrix of the final output. Based on the learned weighting coefficients, the model adaptively fuses the modality information for different emotions. The features are mapped to the target classification dimensions, and the final classification probabilities are output by the Soft max function. $W_{out}$ and $b_{out}$ represent the weight matrix and bias vector of the output layer, respectively.

(11) $$Y=Soft\;\max(H_{4}W_{out}+b_{out})$$

## 4. Experiments

### 4.1. Dataset

Experiments were conducted on both a multimodal dataset and a facial image dataset. To satisfy the requirement of incorporating EEG signals and facial expressions, as well as extracting time–frequency–spatial features within the EEG branch, the DEAP dataset [32] was selected as the multimodal benchmark for validation. The DEAP dataset contains EEG recordings from 32 participants while they watched 40 different video clips, as well as facial video recordings from the first 22 participants during the same viewing sessions. For each sample, participants provided ratings of valence and arousal on a scale from 1 to 9, which were used as the ground truth labels. In this study, these labels were binarized using a threshold of 5. Specifically, ratings greater than 5 were labeled as 1 (high), while ratings less than or equal to 5 were labeled as 0 (low). To ensure consistency across the multimodal data, only the recordings from the first 22 participants, for whom facial videos were available, were included in the analysis. Each participant’s session lasted 63 s, with the initial 3 s designated as preparation time and the remaining 60 s corresponding to the actual task period. The raw signals were downsampled to 128 Hz and bandpass-filtered between 4 and 45 Hz by the dataset providers, with EOG artifacts removed via blind source separation. The 3-s preparation period each trial was divided into six 0.5-s windows. DE features were extracted per channel and frequency band, averaged across the six windows, and subtracted from the corresponding trial DE features to eliminate subject-level resting-state biases. During the 60-s actual task period, the data were divided into 120 segments, and DE features were extracted from each segment without averaging. To ensure consistency between the facial expression data and the EEG signals, facial video frames were sampled every 0.5 s. Consequently, 4800 facial expression images were generated for each participant, resulting in a total of 105,600 images for all 22 participants.

The FER-2013 dataset [33] consists of 35,887 images, each with a resolution of 48 × 48 pixels and stored in a single-channel (grayscale) format. Each image belongs to one of the seven classes: anger, disgust, fear, happiness, sadness, surprise, and neutral. We used all 28,709 training images for model training, validated the model on 3500 validation images, and reported the model accuracy on the 3589 images in the test set.

The CK+ dataset [34] is an extension of the original Cohn–Kanade (CK) facial expression database, incorporating facial expression data from an additional 123 adult participants on top of the original 97 subjects. The participants performed a series of 23 facial displays, including individual AU as well as combinations of AUs. The dataset consists of 593 facial expression sequences from 123 subjects, among which 327 sequences are annotated with explicit emotion labels. Each sequence evolves from a neutral expression to a peak expression, effectively capturing the dynamic progression of facial expressions. The CK+ dataset provides seven discrete emotion categories, including anger, disgust, fear, happiness, sadness, surprise, and contempt. For experimental use, the last three frames closest to the peak expression were extracted from each sequence. CK+ is a relatively small, pose-controlled facial expression dataset. Its inclusion in this study serves as a controlled validation of the MSDAC architecture, rather than as a primary generalization benchmark. The more challenging FER-2013 dataset, comprising 35,887 real-world images captured under unconstrained conditions, provides the principal evidence of the facial branch’s generalization capability.

### 4.2. Experimental Setup

The face and EEG branches are trained independently with the Adam optimizer (learning rate 5 × 10^−4^), cross-entropy loss, and batch sizes of 128 (face) and 32 (EEG). The fusion model is trained with Adam (learning rate 1 × 10^−4^), batch size 32, and 20 epochs. For robust fusion under modality dropout, EEG and face modalities are randomly dropped with probability 0.1 during training. All models are implemented in PyTorch 2.8.0 and trained on NVIDIA GeForce RTX 2080Ti GPU.

To evaluate the effectiveness of the proposed method, a subject-dependent evaluation protocol consistent with Xiao et al. [27] is adopted on the DEAP dataset. Specifically, five-fold cross-validation is applied independently to each subject. The average classification accuracy (ACC) and standard deviation (STD) across the five folds represent the performance of each individual subject, and the mean ACC and STD across all subjects denote the final performance of the method.

### 4.3. Effectiveness of the Multi-Scale Dilated Attention Module

To evaluate the effectiveness of the proposed Multi-Scale Dilated Attention Network in facial emotion recognition, comparative experiments were conducted on the FER-2013 and CK+ datasets against existing approaches, as summarized in Table 1.

The proposed Multi-Scale Dilated Attention–based facial emotion recognition method achieves an average accuracy of 74.1% on the FER-2013 dataset and 99.69% on the CK+ dataset. The experimental results demonstrate that the Multi-Scale Dilated Attention Network exhibits outstanding classification performance in facial emotion recognition. By incorporating the designed multiscale dilated convolution module and the dual-branch attention mechanism, the model effectively captures multi-scale facial Action Unit features and salient local regions, thereby substantially enhancing classification accuracy. Overall, the final experimental results confirm the superior recognition capability of the proposed model.

To verify the parameter efficiency advantage of MSDAC over the Multi-Scale Attention Convolution (MSAC) employing standard convolutions, Table 2 presents a comparative analysis of the parameter counts required by the proposed MSDAC and MSAC architectures.

The experimental results demonstrate that, under identical input dimensions, MSDAC achieves a reduction of approximately 0.13 MB in parameter size and a decrease of 6.33 MB in total storage requirements compared to the MSAC network. These findings indicate that MSDAC is capable of maintaining competitive recognition performance while simultaneously reducing computational complexity.

### 4.4. Ablation Study

To evaluate the contribution of each module within the proposed MSDAC framework, a series of ablation experiments with different configurations was conducted. The D-BA and the MSDC were sequentially removed, and the performance of the resulting variants was compared with that of the complete network on the DEAP dataset. In addition, to further demonstrate the effectiveness of dilated convolution in facial emotion recognition, a Multiscale Attention Convolution (MSAC) variant was designed by replacing the dilated convolution in MSDAC with standard convolutions. The corresponding results are presented in Table 3.

The experimental results on the DEAP dataset demonstrate that the baseline network without D-BA and MSDC achieves valence and arousal accuracies of 92.47% and 91.53%, respectively. With the inclusion of the MSDC module, the accuracies increased to 96.27% (valence) and 94.31% (arousal). When the dilated convolutions in MSDAC are replaced with standard convolutions, the valence and arousal accuracies reach 97.62% and 95.94%, respectively. The best results are attained when both D-BA and MSDC are integrated, yielding accuracies of 98.05% (valence) and 96.15% (arousal). These results demonstrate that the Dual-Branch Attention module enhances the fused feature map, improving the average accuracy by 3.8% for valence and 2.78% for arousal compared with the model without D-BA. The combination of D-BA and MSDC enables more precise capture of critical facial expression cues across multiple scales, resulting in a substantial performance gain of 5.58% (valence) and 4.62% (arousal) over the baseline network. The results in Table 3 prove that the synergy between MSDC and D-BA is not merely additive. The 5.58% performance jump indicates that capturing multi-scale AU correlations is essential for the attention module to correctly highlight emotionally salient regions. This validates our design choice of using dilated convolutions as the structural foundation rather than standard convolutions.

### 4.5. Effectiveness of the Multimodal Emotion Recognition Model

**Figure 4:**
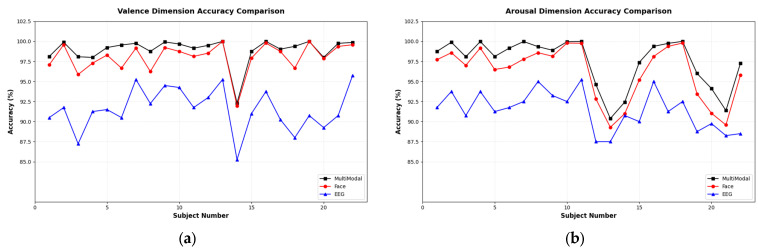
Comparison of Single Modal and Multimodal Emotion Recognition Accuracy for Each Subject on the DEAP Dataset: (**a**) Classification results of Valence emotional dimension; (**b**) Classification results of Arousal emotional dimension.

Figure 4 presents a comparison of single-modal and multimodal emotion recognition accuracy across 22 subjects on the Valence and Arousal dimensions of the DEAP dataset. In the figure, the blue triangular markers represent the EEG single-modal recognition results, the red circular markers represent the facial expression single-modal recognition results, and the black square markers represent the multimodal fusion method recognition results.

Experimental results demonstrate that on the Valence and Arousal dimensions, the EEG single-modal approach achieves average accuracies of 91.53% and 91.42%, respectively, while the MSDAC-based facial expression single-modal approach achieves 98.05% and 96.15%, respectively. In contrast, the proposed multimodal fusion method attains average accuracies of 98.66% and 97.49%, respectively. Compared to the EEG single-modal approach, the multimodal method improves accuracy by 7.13% on Valence and 6.07% on Arousal. Compared to the facial expression single-modal approach, the improvements are 0.61% on Valence and 1.34% on Arousal. To verify whether the performance improvement is statistically significant, we performed a Paired *t*-test comparing our proposed self-learning weight fusion model against the facial expression across all test folds. For the Valence dimension, the analysis yielded *p* = 0.024 (*p* < 0.05). For the Arousal dimension, the analysis yielded *p* = 0.031 (*p* < 0.05). These results confirm that the performance enhancement, though numerically small in high-accuracy scenarios, is statistically significant and not the result of random fluctuations. Despite considerable inter-subject variability observed in single-modal recognition, such as accuracy fluctuations in the EEG modality for subjects 14–15, the multimodal fusion method consistently maintains high and stable recognition accuracy, demonstrating its robustness in handling modal uncertainty and individual differences. These results substantiate the effectiveness of the proposed self-learning weighted decision-level fusion strategy in integrating heterogeneous modal information and significantly enhancing emotion recognition performance.

To further verify the effectiveness of the proposed multimodal emotion recognition framework, we conducted comparative experiments between the proposed multimodal approach, EEG-only and facial-only single modal methods, as well as several existing multimodal emotion recognition techniques. The results are summarized in Table 4.

Among the multimodal fusion strategies evaluated, the integration of EEG and facial expression information exhibits the most competitive performance. Compared with EEG combined with Peripheral Physiological Signals (PPSs) or ECG signals, the fusion of EEG and facial expression features enables more precise identification of emotional states. The proposed method achieves the best performance on the DEAP dataset, further confirming the superiority and effectiveness of the proposed approach.

**Figure 5:**
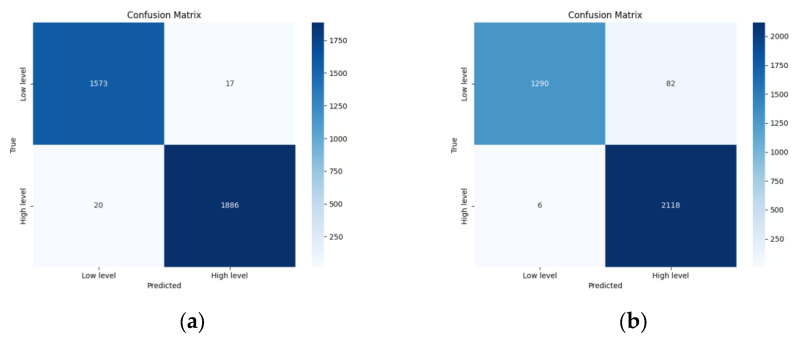
Confusion matrix on the DEAP dataset: (**a**) Confusion Matrix of Valence Dimension; (**b**) Confusion Matrix of Arousal Dimension.

To illustrate the recognition performance of the proposed multimodal emotion recognition model, confusion matrices on the DEAP dataset were plotted, as shown in Figure 5. For the valence dimension, the majority of misclassifications occur when predicting Low valence as High valence, consistent with the known positive bias in DEAP participant ratings. For arousal classification, the confusion matrix similarly suggests that low-arousal samples present greater ambiguity, likely due to the reduced discriminability of EEG spectral features under low-activation states. These observations are consistent with the higher standard deviation observed for EEG single modal arousal recognition compared to the facial modality, indicating that the EEG branch is the primary source of uncertainty in difficult cases.

### 4.6. Effectiveness of the Self-Learning Weight Module

To evaluate the effectiveness of the proposed self-learning weight module, a comparative experiment was conducted on the DEAP dataset against feature level fusion and conventional weighted fusion method, as shown in Table 5. In the feature level fusion, the high-dimensional features from the Face and EEG branches are concatenated before the final classification. In the weighted fusion method, fixed weights are assigned to the decision outputs of different modalities, and the final result is obtained through weighted summation. The formulation is expressed as follows:

(12) $$X_{fused}=kX_{E}+(1-k)X_{F}$$

where k∈[0,1] denotes the fixed fusion weight.

Experimental results demonstrate that feature level fusion achieves accuracies of 94.85% and 93.58% for valence and arousal, respectively. While this represents an improvement over the EEG single modal approach, the performance remains inferior to both the facial single modal branch and the proposed self-learning weighting method. This discrepancy may be attributed to the high heterogeneity between EEG signals and facial features, where direct concatenation at the feature level can lead to significant information interference and the modality collapse phenomenon. The self-learning weighting method improves the average accuracy by 1.41% for valence and 1.02% for arousal compared with the conventional weighted fusion strategy. These findings confirm that the proposed self-learning weight module enables a more effective integration of EEG and facial modalities, thereby leading to a notable enhancement in classification performance. Facial expressions reflect overt emotional states that individuals may consciously regulate, whereas EEG signals originate directly from the central nervous system and capture covert emotional components that facial expressions may not fully convey. Since subjects can suppress facial displays due to social norms but cannot voluntarily control EEG responses, the EEG modality provides critical complementary information on boundary samples where facial expressions are ambiguous or suppressed. This inherent complementarity also explains why the self-learning weight module outperforms fixed-weight fusion, as it dynamically adjusts each modality’s contribution based on per-sample reliability rather than applying a rigid weighting scheme.

## 5. Conclusions

This paper proposes a self-learning multimodal emotion recognition framework based on multi-scale dilated attention to overcome the limitations of existing methods in terms of insufficient facial feature extraction and inflexible multimodal fusion strategies. By integrating the MSDAC network for facial expression modeling with a decision-level fusion mechanism that can self-learn the weights of EEG and facial modalities, the proposed approach can effectively capture fine-grained facial action unit patterns as well as the dependencies between different modalities, thereby improving overall multimodal emotion recognition performance from both intra-modal and inter-modal perspectives. Experiments on the DEAP dataset show that the proposed model achieves recognition accuracies of 98.66% and 97.49% on the Valence and Arousal dimensions, respectively, significantly outperforming single-modal baselines and representative multimodal fusion schemes. These results indicate that achieving high-precision emotion recognition from EEG and facial signals largely depends on powerful feature representations and adaptive relationship modeling, and further confirm the robustness and effectiveness of the proposed framework for multimodal affective computing tasks.

The proposed multimodal emotion recognition framework has broad application prospects. In the field of mental healthcare, the system could serve as a non-invasive auxiliary tool for continuous affective state monitoring, supporting early screening and longitudinal tracking of conditions such as depression, anxiety disorders, and post-traumatic stress disorder, where subtle emotional dysregulation patterns can manifest across both facial and neurophysiological channels. In human–computer interaction (HCI) and adaptive systems, the ability to infer user emotional states in real time could enable more responsive interfaces that adjust content presentation, task difficulty, or interaction style to the user’s current affective condition, improving user experience in applications ranging from intelligent tutoring systems to virtual assistants. As wearable EEG technology continues to mature and facial analysis systems become increasingly deployable on commodity hardware, the clinical and commercial viability of frameworks such as the one proposed here is expected to grow substantially.

Despite the strong performance demonstrated, several limitations should be acknowledged. First, the multimodal evaluation is conducted exclusively under a subject-dependent protocol on the DEAP dataset, which involves only 22 participants with available facial video recordings. The generalizability of the proposed framework to cross-subject or cross-dataset scenarios has not been explicitly validated. The substantial inter-subject variability inherent in EEG signals remains an open challenge for multimodal emotion recognition systems. In future work, the proposed framework could be extended to cross-subject scenarios by incorporating domain adaptation strategies and subject-invariant feature learning methods, and its generalizability could be further validated through Leave-One-Subject-Out (LOSO) evaluation on the DEAP dataset and additional benchmarks. Furthermore, regarding the facial recognition branch, future work may explore the performance of facial recognition methods on larger-scale datasets, and investigate cross-dataset transfer learning strategies to further improve the generalization capability of the model.

## Figures and Tables

**Table 1:** Accuracy comparison of various methods on FER-2013 and CK+ dataset.

Method	FER-2013 (%)	CK+ (%)
DEACN [15]	70.02	98
TF-CNN [13]	64.5	91
MIANet [22]	72.28	95.76
AttenVGG [16]	65.04	99.39
LightExNet [14]	69.7	97.37
MSDAC	74.1	99.69

**Table 2:** Comparison of Parameter Counts Between MSDAC and MSAC on the DEAP Dataset.

Method	Parameters	Input	Total Memory	Accuracy ± Std (%)	
				Valence	Arousal
MSDAC	1.39 MB	0.43 MB	68.02 MB	98.05 ± 0.27	96.15 ± 0.34
MSAC	1.52 MB	0.43 MB	74.35 MB	97.62 ± 0.88	95.94 ± 0.64

**Table 3:** Ablation Study on the DEAP Dataset.

Classification	W/O D-BA + MSDC	W/O D-BA	MSAC	MSDAC
Valence (%)	92.47	96.27	97.62	98.05
Arousal (%)	91.53	94.31	95.94	96.15

**Table 4:** Comparison Between the Proposed Multimodal Method and Other Multimodal Methods.

Method	Modal	Fusion Type	Accuracy ± Std (%)		F1-Score ± Std (%)	
			Valence	Arousal	Valence	Arousal
CMC-Net [9]	EEG + ECG	Feature level	79.62	81.69	75.01	73.51
MM-CRNN [35]	EEG + PPS	Feature level	93.06	91.95	-	-
DCMMER [36]	EEG + Face	Feature level	95.25 ± 3.84	94.89 ± 3.07	94.50 ± 3.97	95.55 ± 3.30
MFEE-Fusion [37]	EEG + Face	Decision level	95.30	94.94	94.53	93.91
3DST-CNN [30]	EEG + Face	Hybrid fusion	96.13	96.19	-	-
EDA-CNN [29]	EEG + Face	Feature level	96.63	97.15	-	-
DG-JCA [38]	EEG + Face	Feature level	92.50 ± 0.41	92.89 ± 0.62	-	-
Ours	EEG		91.53 ± 3.51	91.42 ± 3.47	90.91 ± 3.51	89.83 ± 3.98
Ours	Face		98.05 ± 0.27	96.15 ± 0.34	96.60 ± 0.41	94.08 ± 0.27
Ours	EEG + Face		98.66 ± 0.21	97.49 ± 0.64	97.34 ± 0.95	96.75 ± 1.48

**Table 5:** Validation of Effectiveness for Self-Learning Weight Module.

Method	Accuracy ± Std (%)	
	Valence	Arousal
EEG	91.53 ± 3.51	91.42 ± 3.47
Face	98.05 ± 0.27	96.15 ± 0.34
Feature Level Fusion EEG + Face	94.85 ± 2.65	93.58 ± 2.82
Weighted Fusion EEG + Face	97.25 ± 1.44	96.47 ± 1.94
Self-Learning Weight Fusion EEG + Face	98.66 ± 0.21	97.49 ± 0.64

## Data Availability

The original code and data presented in the study are openly available at https://github.com/yy11z/SL_MER_MSDA (accessed on 13 February 2026).

## Abbreviations

The following abbreviations are used in this manuscript:

EEG	Electroencephalogram
ECG	Electrocardiogram
FER	Facial Emotion Recognition
AUs	Action Units
CNN	Convolutional Neural Network
LSTM	Long Short-Term Memory
DE	Differential Entropy
PPS	Peripheral Physiological Signals
LOSO	Leave One Subject Out
